# Quantitative Phosphoproteomics of Proteasome Inhibition in Multiple Myeloma Cells

**DOI:** 10.1371/journal.pone.0013095

**Published:** 2010-09-29

**Authors:** Feng Ge, Chuan-Le Xiao, Li-Jun Bi, Sheng-Ce Tao, Sheng Xiong, Xin-Feng Yin, Li-Ping Li, Chun-Hua Lu, Hai-Tao Jia, Qing-Yu He

**Affiliations:** 1 Institute of Life and Health Engineering and National Engineering Research Center of Genetic Medicine, Jinan University, Guangzhou, China; 2 Institute of Hydrobiology, Chinese Academy of Sciences, Wuhan, China; 3 National Laboratory of Biomacromolecules, Institute of Biophysics, Chinese Academy of Sciences, Beijing, China; 4 Shanghai Center for Systems Biomedicine, Shanghai Jiaotong University, Shanghai, China; Johns Hopkins School of Medicine, United States of America

## Abstract

**Background:**

The proteasome inhibitor bortezomib represents an important advance in the treatment of multiple myeloma (MM). Bortezomib inhibits the activity of the 26S proteasome and induces cell death in a variety of tumor cells; however, the mechanism of cytotoxicity is not well understood.

**Methodology/Principal Findings:**

We investigated the differential phosphoproteome upon proteasome inhibition by using stable isotope labeling by amino acids in cell culture (SILAC) in combination with phosphoprotein enrichment and LC-MS/MS analysis. In total 233 phosphoproteins were identified and 72 phosphoproteins showed a 1.5-fold or greater change upon bortezomib treatment. The phosphoproteins with expression alterations encompass all major protein classes, including a large number of nucleic acid binding proteins. Site-specific phosphopeptide quantitation revealed that Ser38 phosphorylation on stathmin increased upon bortezomib treatment, suggesting new mechanisms associated to bortezomib-induced apoptosis in MM cells. Further studies demonstrated that stathmin phosphorylation profile was modified in response to bortezomib treatment and the regulation of stathmin by phosphorylation at specific Ser/Thr residues participated in the cellular response induced by bortezomib.

**Conclusions/Significance:**

Our systematic profiling of phosphorylation changes in response to bortezomib treatment not only advanced the global mechanistic understanding of the action of bortezomib on myeloma cells but also identified previously uncharacterized signaling proteins in myeloma cells.

## Introduction

The ubiquitin-proteasome pathway is responsible for proteolysis of eukaryotic cellular proteins related to cell cycle regulation, cell survival, and apoptosis [1]. Inhibition of proteasome activity is a novel therapeutic strategy against cancer cells. Bortezomib (formerly known as PS-341), a cell-permeable boronic acid dipeptide, is a specific inhibitor of the proteasome pathway [2] and received Food and Drug Administration (FDA) approval for the treatment of MM and mantle cell lymphoma [3].

Bortezomib has been reported to trigger pleiotropic signaling pathways in MM cells, including: (a) stabilizing cytoplasmic IκB and blocking NFκB nuclear translocation [4]; (b) activation of stress response proteins such as heat shock proteins Hsp27, Hsp70, and Hsp90 [5]; (c) up-regulation of c-jun NH2-terminal kinase [6]; (d) induction of intrinsic cell death pathway [7]; (e) activation of extrinsic apoptotic signaling through Bid and caspase-8 cleavage [8]; (f) impairment of DNA repair machinery via inactivation of DNA-dependent protein kinase [9]; (g) down-regulation of mitogen-activated protein kinase and phosphatidylinositol 3-kinase/Akt signaling pathways [10]; and (h) down-regulation of the p44/42 MAPK signaling cascade [11]. All these signaling events may collectively contribute towards the overall anti-MM activity of bortezomib. However, the exact number and identity of cellular signaling events involved in proteasome inhibition and the mechanisms underlying the associated apoptotic response in MM cells remain to be elucidated.

Elucidation of cellular signaling networks requires methodologies for large-scale quantitative phosphoproteomic analysis that can reveal dynamic system-wide changes in protein phosphorylation. Recent technological advances in mass spectrometry-based proteomics have enabled us to make a large-scale identification of signaling molecules through the enrichment of phosphorylated proteins or peptides [12], [13]. One of the most widely used and well known strategies currently used in phosphoproteomic studies is stable-isotope labelling by amino acids in cell culture (SILAC). Although introduced relatively recently, SILAC has been used extensively in the proteomics community [14]. With SILAC, the entire proteome of a given cell population is metabolically labeled by heavy, non-radioactive isotopic variants of amino acids, thus making it distinguishable by MS analysis [15], [16]. Thereafter, two or more distinctly SILAC-labeled cell populations can be mixed and analyzed in one MS experiment, allowing accurate quantization of proteins from the different cellular states. By coupling with a phosphoprotein or phosphopeptide enrichment method, such as titanium dioxide (TiO_2_) [17], strong cation exchange (SCX) [18], or the two in combination [19], SILAC has been widely applied to profile dynamic phosphorylation changes in signal transduction [20], [21].

In this study, we investigated the differential MM phosphoproteome upon proteasome inhibition by using SILAC in combination with phosphoprotein enrichment and LC-MS/MS analysis. Many potential novel signaling proteins and associated signaling pathways were confidently identified. Our further functional results indicated that perturbations in stathmin phosphorylation play a significant functional role in mediating apoptosis in MM cells exposed to bortezomib and the bortezomib-induced changes in the MT stabilization can be attributed to the bortezomib-induced phosphorylation of stathmin. By correlating the phosphoproteomic data with functional studies, the current results provided novel insights into the mechanisms of bortezomib actions in MM cells.

## Results

### Quantitative Phosphoproteomic Analysis of Proteasome Inhibition in Myeloma Cells

**Figure 1:**
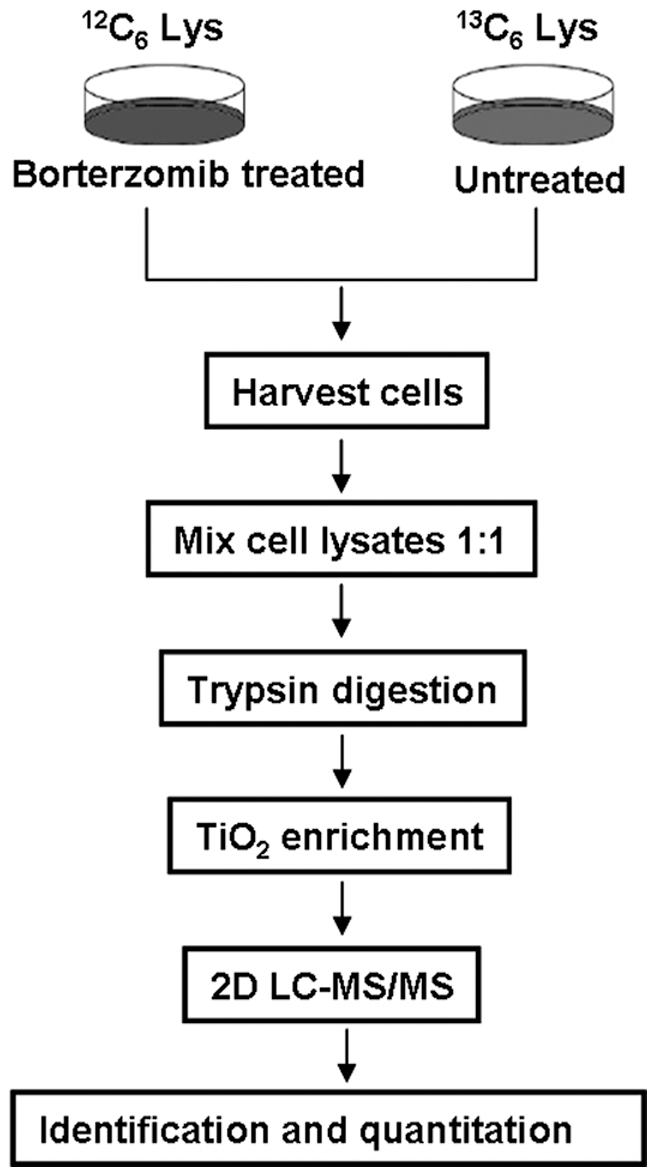
Schematic diagram of the experimental procedures. The application of SILAC in differential phosphoproteomic profiling of myeloma cells U266 treated by bortezomib.

**Figure 2:**
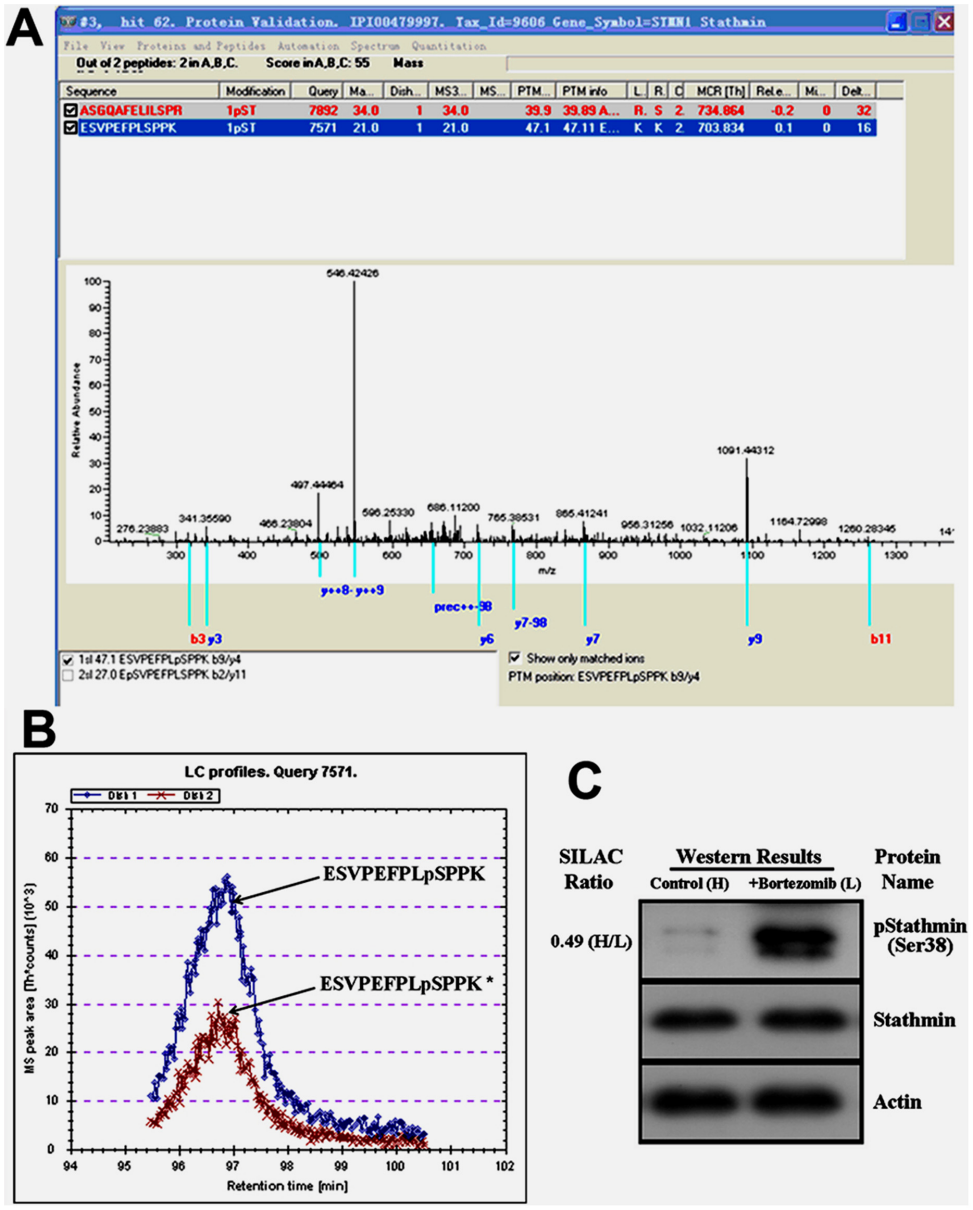
Identification and quantization of Ser38 phosphorylation of stathmin. (A) Representative MS/MS spectrum of phosphopeptide ESVPEFPLpSPPK. (B) Representative chromatogram of ESVPEFPLpSPPK. Ion chromatograms of [^12^C_6_] lysine- and [^13^C_6_] lysine-containing peptides identified by MS/MS were extracted from the series of MS scans by using MSQuant. (C) Western blot analyses using specific antibodies showed that phosphorylation of stathmin at Ser38 was increased, whereas steady-state stathmin remained almost unchanged upon bortezomib treatment.

To obtain a global view of the changes of protein phosphorylation in bortezomib-treated myeloma cells, we compared the phosphoproteome of U266 cells treated with or without bortezomib. The workflow is outlined in Figure 1. Cells in normal medium (light culture) were treated with bortezomib, and cells grown in medium containing stable isotopes (heavy culture) were treated with vehicle. These two populations of cells were lysed, mixed at a 1∶1 ratio, and subjected to TiO_2_ purifications followed by LC-MS/MS analysis. After LC-MS/MS analysis on the enriched phosphopeptides, all MS/MS spectra were searched, respectively, against the forward and reversed human protein sequence databases to estimate rates of false-positive matches. Search results were filtered based on peptide score of MASCOT and PTM score. In total 1024 phosphopeptides (redundant) from the target database passed our criteria, allowing 14 decoy matches. The phosphopeptide false-positive rate was therefore estimated to be 1.4%. Multiple filtering criteria were established to validate search results. For each of the phosphorylated peptides identified in this work, peptide sequences were manually confirmed. After validation we identified 418 unique phosphorylation sites from 244 unique phosphopeptides corresponding to 233 protein groups. This entire dataset is provided as Table S1 in which hyperlinks are built up to view all the MS/MS spectra. Using the PTM score, we could localize the phosphor groups with high confidence (Class I phosphorylation sites) in 197 cases (Table S1), indicating that the phosphorylation sites detected by current strategy are of high confidence. Figure 2A shows a representative MS/MS spectrum for a phosphosite-containing peptide in the detection, and all other MS/MS spectra are available via the hyperlinks in the Table S1.

To quantify the phosphorylation change for each phosphopeptide, we used the MSQuant software to calculate area ratio, defined as the ratio of the “heavy” peak area over the “light” peak area in the chromatogram (Fig. 2B). All together, we have achieved quantification of 259 unique phosphorylation sites from 154 unique phosphopeptides corresponding to 132 protein groups. Based on a predefined threshold of 1.5-fold change, 131 phosphosites from 75 unique phosphopeptides corresponding to 72 proteins showed a 1.5-fold or greater change as listed in Table S2. In other words, 31% of the total phosphosites detected showed significant alteration after bortezomib treatment, suggesting that dysregulated phosphorylation may play an important role in bortezomib-induced apoptosis.

### Validation of Differential Expressed Phosphoproteins

To further confirm the results from the quantitative phosphoproteome analysis, we chose stathmin for Western blotting verification using an anti-phospho-Ser38 stathmin antibody. As shown in Figure 2C, SILAC results were very much consistent with Western blotting analysis for this protein. Upon bortezomib treatment, phosphorylation of stathmin at Ser38 increased, whereas steady-state stathmin remained almost unchanged in Western blotting verification.

### Functional Categories and Biological Interaction Networking of Bortezomib-Regulated Phosphoproteins

**Figure 3:**
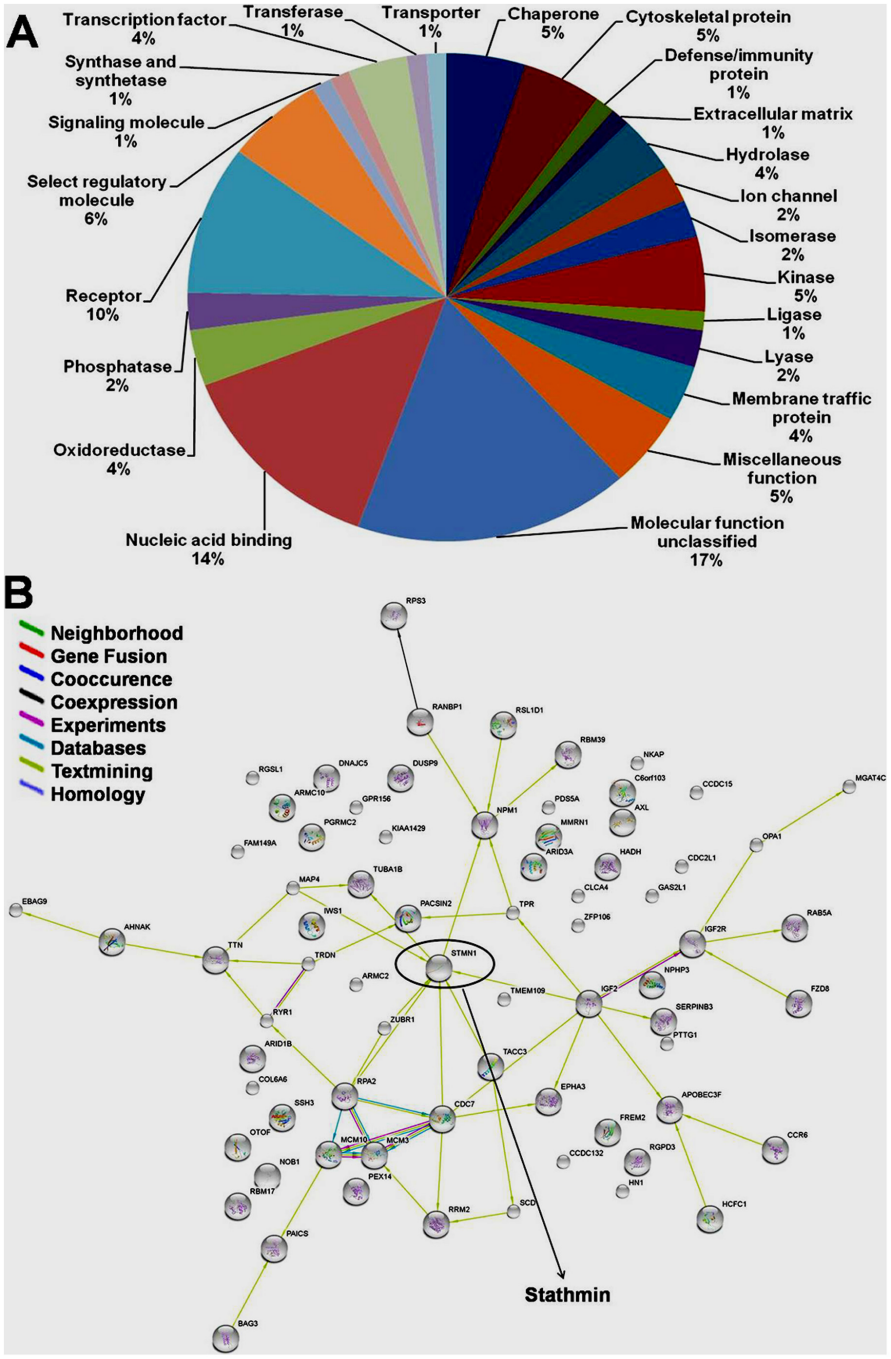
Bioinformatic analysis of identified DEPPs. (A) Pie chart representations of the distribution of identified DEPPs according to their molecular functions. Categorizations were based on information provided by the online resource PANTHER classification system. (B) The protein interaction network of the identified DEPPs. The network was mapped using the STRING system (http://string.embl.de/) based on evidence with different types. Different line colors represent the types of evidence for the associations, which are shown in the legend.

To better characterize bortezomib-regulated phosphoproteins, we classified all the differentially expressed phosphoproteins (DEPPs) into 23 functional categories according to the PANTHER system. These proteins are implicated in a broad range of cellular activities (Fig. 3A). Next to the unclassified proteins (17%), proteins involved in nucleic acid binding account for the second largest portion (14%). There are also a significant number of proteins involved in receptor (10%), regulatory molecule (6%), and kinase (5%). Previous studies indicated that bortezomib exerts its anticancer function by inhibiting protein degradation in cancer cells [22]. In this connection, many bortezomib-regulated phosphoproteins found in current study were nucleic acid binding (14%) and transcription factors (4%), as shown in Figure 3A. These data suggest that the cancer-inhibitory effect of bortezomib may also rely on its regulatory role in mRNA transcription.

Table S2 only shows a list of all borterzomib regulated phosphoproteins in MM cells. It lacks the biochemical context. To create significance out of otherwise static proteomic data, we constructed an interaction networking of the DEPPs (Fig. 3B). The DEPPs were linked by various evidences based on neighborhood analysis, experimental results or text-mining. These relationships are color coded, and the legends are provided next to the map. This does not mean that all the interactions took place within a single spatial and temporal situation, but it enables the identification of central nodes in these DEPPs. Notably, stathmin was a hub in this network, suggesting that stathmin may play an important role in mediating bortezomib-induced apoptosis in MM cells. Some proteins remained orphans because there is insufficient information in the database to link them to other proteins in the network.

### Bioinformatics Analysis

To further investigate the reliability of the results, PhosphoSite (http://www.Phosphosite.org) was used to distinguish known phosphorylation sites from novel phosphorylation sites. Among the identified 418 phosphorylated sites, 51% were also reported by others previously (for details, see Table S3). In other words, many of the phosphorylation sites were also determined by researchers with other cancer cells, further demonstrating that the phosphorylation sites detected by current strategy are reliable.

To predict the kinase substrate relationships from the dataset, the computer algorithm SCANSITE was used. SCANSITE makes use of peptide library phosphorylation data to predict substrates recognized by specific kinases. Tables S4 & S5 show the results of phosphopeptides that were identified in this study and were predicted to be associated with a kinase binding motif by SCANSITE at the different stringency levels. It was found that most of the phosphorylation sites determined in this study were phosphorylated by acidophilic serine/threonine kinase and proline-dependent serine/threonine kinase. Notably, we found fifteen sites that can be phosphorylated by the Casein Kinase 2 (CK2). CK2 is a constitutively active protein kinase implicated in cellular transformation and the development of tumorigenesis [23]. Aberrantly active in MM cells, CK2 controls the cell survival [24]. Our SCANSITE analysis suggests that CK2 may play an important role in bortezomib-induced apoptosis and may represent a potential target in MM therapy.

### Regulation of Stathmin Phosphorylation by Bortezomib

**Figure 4:**
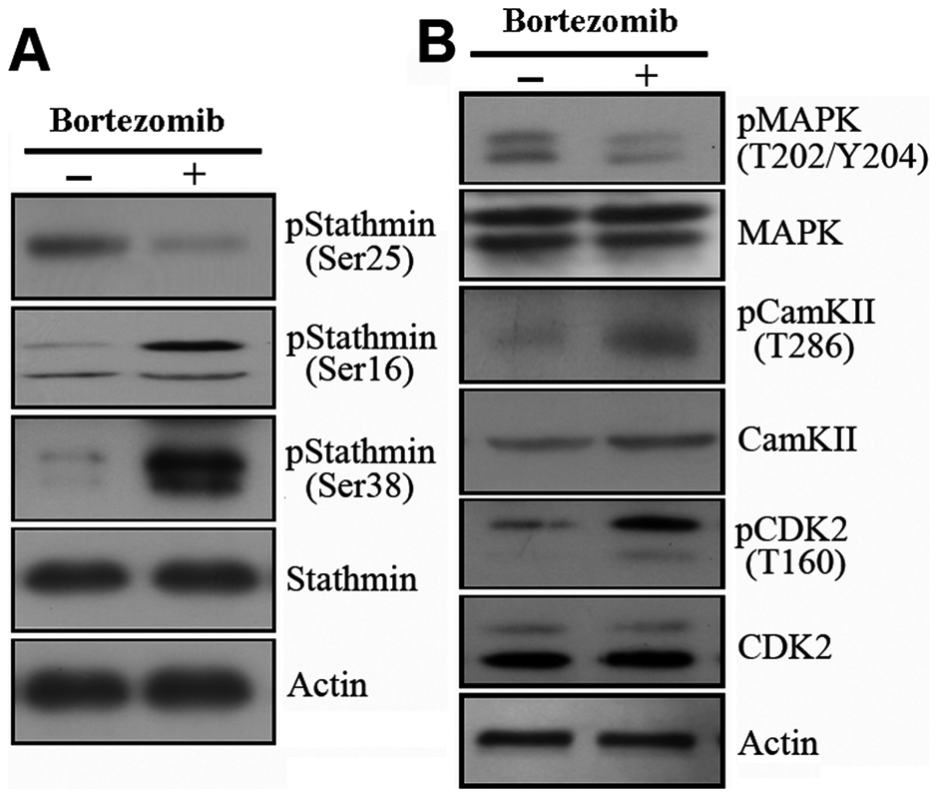
Phosphorylation pattern of stathmin under bortezomib treatment. (A) Upregulation of the phosphorylation at Ser16 and Ser38 and down-regulation of the phosphorylation at Ser25 were observed in bortezomib-treated U266 cells. (B) Activation of kinases targeting these residues (CaMKII, CDK2, and MAPK) was studied by Western blot using specific antibodies. Equal protein loading was assessed with actin.

SILAC phosphoproteomic analyses and Western blotting revealed an increase of phosphorylation of stathmin at Ser38 and the unchanged steady-state stathmin in U266 cells upon proteasome inhibition (Fig. 2). It has been reported that the functional alteration of stathmin resulting from specific phosphorylation events may be involved in the process of apoptosis induced by proteasome inhibitors in proliferating cells [25]. To elucidate the differential phosphorylation of stathmin isoforms, Western blotting was performed to analyze the phosphorylation pattern of stathmin in bortezomib-treated U266 cells using stathmin antibody and specific phospho-antibodies against three known phospho-sites (Ser16, Ser25, Ser38) [26]. As shown in Figure 4A, in accordance with SILAC results, phosphorylation of stathmin at Ser38 was increased whereas steady-state stathmin remained unchanged. Furthermore, phosphorylation of stathmin at Ser16 was increased whereas Ser25 was decreased after bortezomib treatment (Fig. 4A).

It has also been reported that Ser16 is a target for calmodulin-dependent protein kinases (CamKII), Ser25 is specifically phosphorylated by mitogen-activated protein (MAP) kinase, and Ser38 is a target for cycline-dependent kinase-2 (CDK2) [26]. To test the activation status of upstream kinases catalyzing the incorporation of phosphoryl groups to each of these residues, specific antibodies against active forms of CaMKII, MAPK and CDK2 (responsible for the stathmin phosphorylation on Ser16, Ser25 and Ser38, respectively) were used. In accordance with the increase in p-Ser16 and p-Ser38 of stathmin in bortezomib-treated cells, a parallel activation of CamKII and CDK2 was detected after bortezomib treatment (Fig. 4B). At the same time, the decrease in phosphorylation levels of Ser25 was correlated with the inactivation of MAPK, a critical kinase for cell survival.

Hence, these results suggest that the regulation of the phosphorylation profile of stathmin at the level of residues Ser16, Ser25, and Ser38 may participate in the response of myeloma cells to proteasome inhibitors, and that stathmin is a target for multiple protein kinases, which are regulated by multiple signal transduction cascades.

### Mutation of Stathmin Phosphorylation Site Decreases Sensitivity to Bortezomib and Influences Tubulin Polymerization

**Figure 5:**
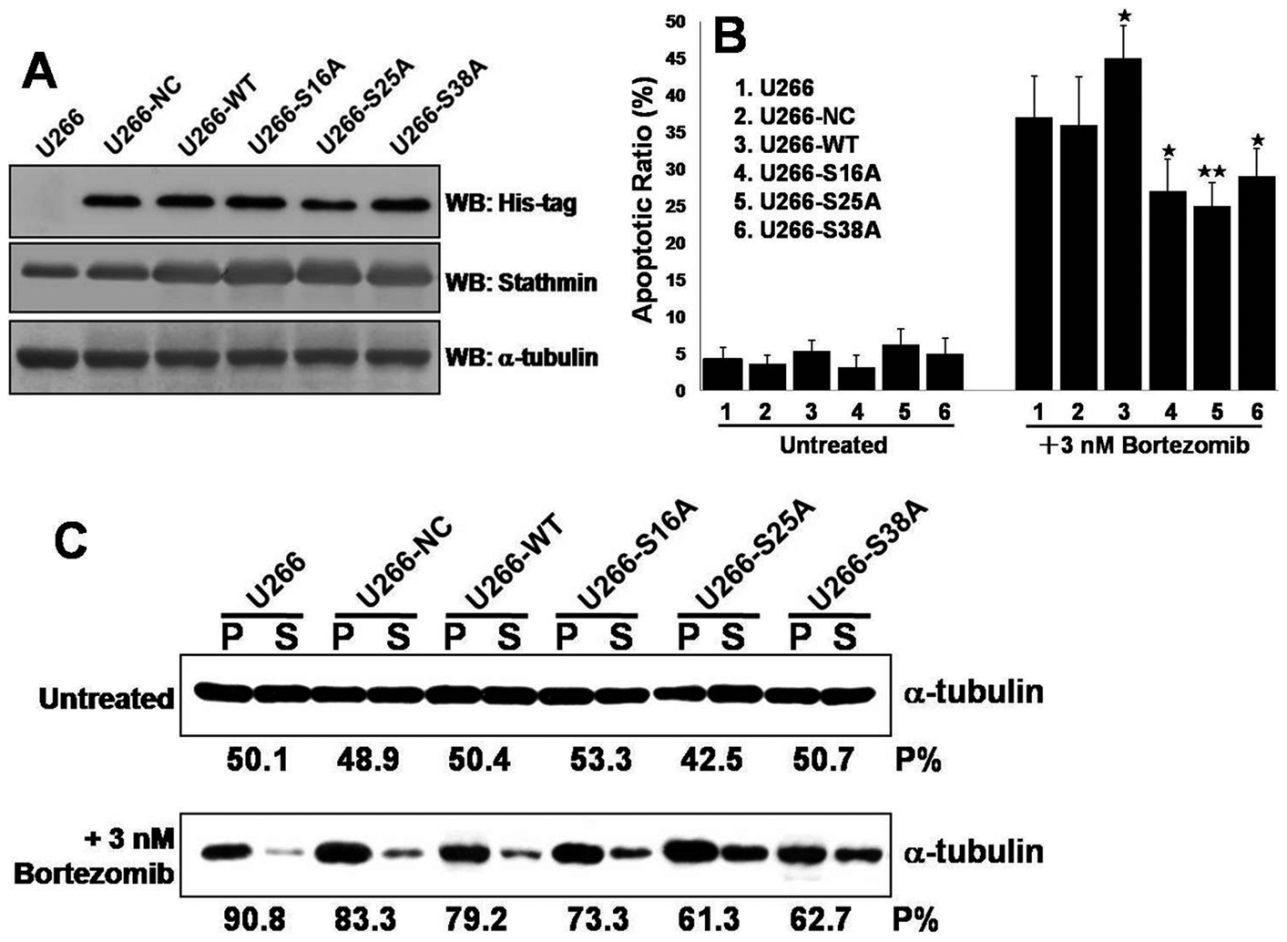
Mutation of stathmin phosphorylation sites decreases sensitivity to bortezomib and influences tubulin polymerization. (A) U266 cells were stably transfected with HA-tagged wild type (WT) and mutant (S16A, S25A, S38A) stathmin constructs or its empty vector (NC). (B) Cells were exposed to 3 nM bortezomib for 24 hr, after which the percentage of apoptotic cells was determined by Annexin V/PI staining and flow cytometry. Results represent the means (± SD) for 3 separate experiments performed in triplicate. (* *P*<0.05 and ** *P*<0.01). (C) Analysis of tubulin polymerization in U266 or derived cells with or without bortezomib treatment. Lysates from these cells were obtained from cells either treated or not with bortezomib at the indicated concentration. Lysates were separated into polymerized (P) or soluble (S) fractions and aliquots of equal volume were separated by SDS-PAGE, the blots probed with anti α-tubulin and the percent of polymerized tubulin calculated for each ‘P’ and ‘S’ pair.

The functional significance of stathmin phosphorylation in the response of MM cells to bortezomib was then examined more rigorously. To this end, stable U266 cell clones overexpressing the stathmin wild-type (U266-WT) or mutants (U266-S16A, U266-S25A and U266-S38A) were generated using His-tagged constructs. Figure 5A shows the expression of His-tagged target proteins as well as stathmin expression in these cells. The complete methods and characterization in terms of proliferation, cell cycle, colony-forming efficiency (CFE) and apoptotic ratio of these cells are described in the Supplemental Data S1. We observed that all these cells had a comparable growth pattern and CFE, similar proportions of cells in G1, S, and G2 plus M phases and similar apoptotic ratio (Table S6). However, there were significant differences between these cells with regard to their sensitivity to bortezomib treatment. As shown in Figure 5B, cells transfected with wild-type stathmin (U266-WT) exhibited significant increase in bortezomib-induced cell death compared with parental cells (U266). In contrast, cells transfected with mutant stathmin (U266-S16A, U266-S25A or U266-S38A) were significantly less sensitive to bortezomib lethality than U266 cells (*P*<0.05 for U266-S16A and U266-S38A or *P*<0.01 for U266-S25A, respectively) (Fig. 5B).

It has been reported that phosphorylation turns off the microtubule destabilizing activity of stathmin [27], [28] and that proteasome inhibitors increase tubulin polymerization and stabilization in myeloma cells [29]. We thus tested whether bortezomib actually induced changes in the polymerization status of the MTs in these cells. Indeed, when these cells were treated with bortezomib for 24 hours, we observed an increase in the amount of tubulin in the polymerized ‘P’ fraction as compared with untreated cells (Fig. 5C). The baseline proportion of α-tubulin in the polymerized fraction ranged from ∼43% to 52%, while the polymerized proportion observed after bortezomib treatment was ∼60–90% (Table 1).

**Table 1:** Percent of polymerized tubulin in U266 and derived cells treated with bortezomib.

Cell	% Polymerized tubulin average ± S.D.		*p*-value	
	No treatment	3 nM bortezomib	*p*1*	*p*2*
U266	50.5±3.2	90.5±9.2	<0.001	-
U266-NC	49.1±6.7	83.2±7.8	0.003	0.075
U266-WT	51.2±4.6	78.6±5.5	0.005	0.042
U266-S16A	52.5±3.9	74.1±7.6	0.007	0.015
U266-S25A	43.6±8.9	60.8±5.1	0.011	0.003
U266-S38A	51.6±7.7	61.8±8.5	0.047	0.004

*p1: 3 nM bortezomib treated *vs.* No treatment.

*p2: U266 derived cells *vs.* U266 cells with bortezomib treatment.

To investigate whether these bortezomib-induced changes in the tubulin polymerization were mediated by phosphorylation of stathmin, we examined the tubulin polymerization in stable U266 clones that overexpressing WT stathmin and the phosphorylation site-deficient stathmin mutants S16A, S25A and S38A. As shown in Figure 5C, by comparing with U266 cells, overexpression of WT stathmin and phosphorylation site-deficient mutants resulted in a significant decrease in the percent of polymerized tubulin following treatment with bortezomib (Fig. 5C and Table 1).

Thus, our findings support the notion that bortezomib induces tubulin polymerization and stabilization through the mediation by phosphorylation of stathmin and this may be a contribution factor to the mechanism of proteasome inhibition and toxicity in MM cells.

## Discussion

Quantitative phosphoproteomic approaches offer great promise for rapid progress in the analysis of drug targets or mechanism of action, especially when combined with traditional biochemical approaches presently used for studying individual proteins [30]. Moreover, the ability to simultaneously measure changes in phosphorylation state of many proteins in a single experiment can provide unique information needed for quantitative modeling of signaling pathways.

In the present study, we used a combined strategy that comprised phosphoprotein enrichment, SILAC, and LC/MS analysis to profile the differential phosphoproteome in bortezomib-treated MM cells. A total of 72 phosphoproteins were found to have a 1.5-fold or greater change upon bortezomib treatment (Table S2). According to their change pattern, these DEPPs can be categorized into up- (<0.75) or down- (>1.5) regulated groups (Table S2). Notably, in comparison with the number of down-regulated DEPPs, many more proteins are up-regulated in this process (70 versus 5). This unbalanced pattern has also been revealed by previous *in vitro* biochemical studies [22]. Many studies have demonstrated that bortezomib can trigger pleiotropic signaling pathways and suppress the proteasomal degradation of multiple phosphorylated singnaling molecules [8], [10], [31]. Therefore, we speculate that the up-regulation of phosporylation of these DEPPs may constitute one of the mechanisms of bortezomib-induced apoptosis in MM cells. The PANTHER classification system also revealed that the DEPPs implicated in a variety of molecular functions, such as nucleic acid binding, receptor, regulatory molecule and so on (Fig. 3A), clearly showing that in addition to targeting proteins involved in apoptotic pathways, bortezomib also altered multiple signaling pathways to induce its anti-cancer effects.

In particular, as suggested by protein network analysis (Fig. 3B), stathmin may play a central role in mediating bortezomib-induced apoptosis in MM cells. Stathmin (also termed as p19, 19K, p18, prosolin, and Op18) is a ubiquitous 19 kDa cytosolic phosphoprotein that is highly expressed in a wide variety of cancers, including a subset of leukemias and breast carcinomas and is a key regulator in the control of proliferation and cell cycle [32], [33]. Stathmin is a phosphorylation responsive regulator of microtubule (MT) dynamics that increases the catastrophe rate of MTs (depolymerization or shrinkage phase of individual MTs) in a dose-dependent manner [34]. Four Ser residues, Ser16, Ser25, Ser38, and Ser63 in stathmin are subjects for phosphorylation in intact cells [26].

The regulation of stathmin phosphorylation is complex and, in all likelihood, multifactorial. For example, phosphorylations at all four Ser residues fluctuate during the cell cycle and CDK2 has been identified as the kinase system involved in cell cycle-regulated phosphorylation of Ser38. Besides, three distinct protein kinases have been identified to phosphorylate stathmin in response to external signals. These kinases are members of the MAPK family that phosphorylates Ser25, cyclic AMP-dependent protein kinase (PKA) that phosphorylates Ser63 and CamKII that phosphorylates Ser16 [26]. Because the kinases acting at these residues are distinct and may be functioning through different pathways, it is reasonable to speculate that regulation of stathmin phosphorylation can be achieved by multiple pathways, thus providing the cell with a finely tunable mechanism for controlling microtubule assembly and dynamics in relation to its needs.

In the present study, the role of stathmin and its phosphorylation in bortezomib-induced cell death was further investigated by overexpression of the WT stathmin and phosphorylation site-deficient stathmin mutants S16A, S25A or S38A in myeloma cells. Overexpression of WT stathmin significantly increased bortezomib-induced cell death. On the contrary, overexpression of the phosphorylation site-deficient stathmin mutants S16A, S25A and S38A significantly decreased, but did not block, bortezomib-induced cell death. Importantly, increased levels of tubulin polymerization were observed upon bortezomib treatment in cells overexpressing WT or mutant stathmin, but to a lesser extent than parental U266 cells (Fig. 5C, Table 1). There are several possible explanations as to why bortezomib-induced cell death is not completely blocked by the stathmin mutants. First, endogenous WT stathmin is still present, and thus the mutant protein has to compete with the WT protein. Second, bortezomib can also modulate the activity of other MT regulatory proteins that may also contribute to the cell death [29] . Furthermore, the MT system is not the only event involved in bortezomib-induced cell death in myeloma cells [22], [31]. This suggests that bortezomib-induced cell death is the result of a concerted series of events.

Therefore, we conclude that bortezomib-induced phosphorylation of stathmin promotes cell death and that phosphorylation on Ser16, Ser25 and Ser38 is necessary for this process. Furthermore, the bortezomib-induced changes in the MT stabilization can be attributed to the bortezomib-induced phosphorylation of stathmin, and MT stabilization is in fact responsible for the bortezomib-induced cell death-promoting activity of phosphorylated stathmin.

Another protein of interest uncovered in this study is BCL2-associated athanogene 3 (BAG3). In the current study, BAG3 was found to have increased phosphorylation at Ser377 upon bortezomib treatment. BAG3 belongs to the evolutionarily conserved BAG family of proteins that were originally isolated based on their ability to interact with the anti-apoptotic protein Bcl-2 [35], [36]. It is involved in a wide variety of cellular processes, including cell survival, cellular stress response, apoptosis and virus replication [16], [37], [38]. Recent evidence implicates an additional function of BAG3 in the regulation of the autophagy pathway. These findings indicate that autophagosome formation and turnover may depend on BAG3 and that BAG3 can stimulate autophagy processes [39], [40]. Autophagy is a major intracellular degradation system. Unlike the ubiquitin-proteasome system (UPS), autophagy is mainly responsible for the degradation of long-lived proteins and subcellular organelles [41], [42]. Autophagy plays important roles in development, cellular homeostasis and cell survival and is frequently activated in tumor cells exposed to chemotherapy or radiation and confers therapeutic resistance [43], [44]. The UPS and autophagy have been viewed as distinct degradation systems, but recent studies suggested that they are functionally coupled and that suppression of the proteasome promotes autophagy [45], [46]. However, the functional connection and the inter-regulation between the two systems are not well understood.

Importantly, Zhu *et al.* has shown that proteasome inhibition activates autophagy through a phosphorylation of eIF2α-dependent mechanism to eliminate protein aggregates and alleviate proteotoxic stress [46]. However, their results also demonstrated that complicated mechanisms are involved in proteasome inhibition-mediated autophagy activation and the phosphorylation of eIF2α only partially accounts for this activation [46]. Thus, it is possible that the complexity of the process may be much higher than presently envisaged, and that other proteins may be as important in controlling autophagy activation as eIF2α.

Based on the critical role of BAG3 in the stimulation of the autophagy pathway, it is tempting to suggest that bortezomib-induced phosphorylation of BAG3 might play an important role in autophagy activation. Therefore, the increase in BAG3 phosphorylation is likely part of the cell's response to bortezomib treatment and appears to represent a novel mechanism with a link between the two protein degradation systems. This speculative idea, however, is not yet supported by the current experimental data, and further investigations are undergoing to determine the functional implication of BAG3 in coordination between the proteasome and autophagy.

In summary, we have, for the first time, performed quantitative phosphoproteomics to study the effects of bortezomib on MM cells. The current results expand the list of bortezomib-targeted phosphoproteins and their phosphorylation sites. Especially, our functional studies indicated that bortezomib-induced phosphorylation of stathmin promotes cell death and that the bortezomib-induced changes in the MT stabilization can be attributed to the bortezomib-induced phosphorylation of stathmin. Our comprehensive study of phosphorylation regulation in proteasome inhibition in MM cells may serve as a valuable resource for future research in the field and thus advance the general mechanistic understanding of bortezomib in MM.

## Materials and Methods

### Cell Culture and Metabolic Labeling

The human myeloma cell line U266 was purchased from American Type Culture Collections (Rockville, MD). Myeloma cells were routinely maintained in RPMI 1640 supplemented with 1% penicillin/streptomycin, 1 mmol/L L-glutamine, and 10% fetal bovine serum at 37°C, 5% CO_2_ in air. Bortezomib was provided by Millennium Pharmaceuticals (Cambridge, MA). To differentially label bortezomib-treated and -untreated U266 cells, the SILAC Protein Quantitation Kit (Pierce Biotechnology, Rockford, USA) was used according to the manufacturer's instruction. In brief, cells were grown in SILAC RPMI 1640 Medium (Pierce Biotechnology, Rockford, USA) containing 10% v/v dialyzed FBS, and either 0.1 mg/mL heavy [^13^C_6_] or light [^12^C_6_] L-lysine (Pierce Biotechnology, Rockford, USA). To ensure full incorporation of the heavy and light labeled amino acids, cells were grown for at least six cell doublings prior to analysis. U266 cells were treated with 3 nM bortezomib for 24 h, according to the half-maximal inhibitory concentration (IC50) measured by Hideshima *et al* [11]. After treatment, cells were washed three times with ice-cold washing buffer (10 µM Tris–HCl, 250 µM sucrose, pH 7.0) and transferred to a clean 1.5 mL Eppendorf tube. Cells were lysed with RIPA lysis buffer (50 mM Tris-HCl, 150 mM NaCl, 0.1% SDS, 1% NP-40, 0.5% sodium deoxycholate, 1 mM PMSF, 100 mM leupeptin, and 2 mg/mL aprotinin, pH 8.0). Cellular debris was removed by centrifugation for 30 min at 13, 200 g and at 4°C. Protein concentrations were measured in duplicate using RC DC protein assay (BioRad, Hercules, CA, USA) and confirmed by SDS-PAGE.

### Phosphopeptide Enrichment using TiO_2_

Phosphopeptide enrichment was performed as previously described [17]. Briefly, equal amounts of proteins from untreated (^13^C_6_-lysine) and bortezomib-treated (^12^C_6_-lysine) U266 cells were mixed (1 mg in total) and subjected to disulfide reduction with 10 mM DTT (37°C, 3 h) and alkylation with 20 mM iodoacetamide (room temperature, 1 h in dark). The protein mixtures were mixed with four volumes of ice-cold acetone to precipitate proteins. Precipitated proteins were collected by centrifugation and washed with ethanol two times. The pellet was re-dissolved in 50 mM ammonium bicarbonate and then digested with sequencing grade modified trypsin (1∶25 w/w) (Promega, Madison, WI) at 37°C for 20 h and then quenched by addition of TFA to a final concentration of 0.5%. The digests were evaporated to about 20 µL in SpeedVac centrifuge. The phosphopeptides from digested peptides were enriched by using Phosphopeptide Enrichment TiO_2_ Kit (Calbiochem, San Diego, CA) according to the manufacturer's instruction with slight modifications. Briefly, the tryptic digest was dried, re-dissolved in 200 µL TiO_2_ Phosphobind buffer containing 50 g/L 2,5-dihydroxybenzoic acid and then mixed with 50 µL TiO_2_ Phosphobind Resin. After 30 min incubation, the supernatant was discarded, and TiO_2_ was washed three times with the wash buffer. After that, 30 µL elution buffer was added two times to elute the phosphopeptides. The elutions were combined and acidified with 5 µL of 10% formic acid for SCX-LC–MS/MS analysis. All the buffers and the phosphopeptides purification resin were provided in the kit by the manufacturer.

### Peptide Analysis by LC-MS/MS Approach

The enriched phosphopeptides were analyzed with a Finnigan Surveyor HPLC system coupled online with a LTQ-Oribitrap XL (Thermo Fisher Scientific, Waltham, MA) equipped with a nanospray source. The phosphopeptides were firstly loaded on a strong cation exchange (SCX) column using an autosampler, and the peptides were eluted by NH_4_Cl with different concentrations (1 mM, 10 mM, 100 mM, 200 mM, 1 M). Then, each fraction peptide was respectively loaded onto a C18 column (100 µm i.d., 10 cm long, 5 µm resin from Michrom Bioresources, Auburn, CA) using an autosampler. Peptides were eluted during a 0–35% gradient (Buffer A, 0.1% formic acid, and 5% ACN; Buffer B, 0.1% formic acid and 95% AcN) over 90 min and online detected in LTQ-Orbitrap using a data-dependent method [47]. The general mass spectrometric conditions were: spray voltage, 1.80 kV; no sheath and auxiliary gas flow; ion transfer tube temperature, 200°C. Ion selection thresholds were: 1000 counts for MS^2^ and 500 counts for MS^3^. An activation q = 0.25 and activation time of 30 ms were applied in MS^2^ acquisitions. The mass spectrometers were operated in positive ion mode, employing a data-dependent automatic switch between MS and MS^2^ acquisition modes. For each cycle, one full MS scan in the Orbitrap at 1×10^6^ AGC target was followed by five MS^2^ in the LTQ at 5000 AGC target on the five most intense ions. Selected ions were excluded from further selection for 90 s. Maximum ion accumulation time was 500 ms for full MS scans and 100 ms for MS^2^ scans. All MS/MS spectra were collected using normalized collision energy (a setting of 35%), an isolation window of 3 *m/z*, and 1 micro-scan. The resolution used in the MS step in the Orbitrap is 60000. An extra DDNL (data-dependent neutral loss) MS^3^ method was applied for phosphopeptide detection [17]. MS^3^ was triggered if a neutral loss peak at −98.0, −49.0, −32.7 or −24.5 Da was observed in the MS^2^ and that peak was one of the three most intense ions of the MS^2^ spectra. Application of mass spectrometer scan functions and HPLC solvent gradients were controlled by XCalibur data system (Thermo Fisher Scientific, Waltham, MA).

### Phosphopeptide Identification, Validation and Quantification

Peak lists for the database search were produced in the Mascot generic format using BioWorks 3.3.1 (Thermo Finnigan, San Jose, CA) and DTASuperCharge V 1.31 (SourceForge), and the derived peak lists were searched using the Mascot 2.2.04 search engine (Matrix Science, London, UK) against a real and false IPI human database (V3.56, including 153, 078 protein entries). The following search criteria were employed: full tryptic specificity was required; two missed cleavages were allowed; Carbamidomethylation was set as fixed modification, whereas Oxidation (M), Phospho (ST), and Phospho (Y) were considered as variable modifications. Precursor ion mass tolerances were 10 ppm for all MS acquired in the Orbitrap mass analyzer, fragment ion mass tolerance was 0.5 Da for all MS^2^ spectra acquired in the LTQ. Mass spectra of identified phosphopeptides with peptide score >10 were further processed and validated with the MSQuant 1.5 software for post-translational modification (PTM) score analysis [48]. Two filters criteria for phosphopeptide identification were applied: 1) Peptide score threshold was 17; 2) The total threshold of PTM score and peptide score was 36. All fragmentation spectra were manually verified using the criteria as described by Mann et al [48]. For phosphopeptides with multiple potential phosphorylation sites, the probabilities for phosphorylation at each site were calculated from the PTM scores as described [48]. Phosphorylation sites that were occupied with probability >0.75 were reported as class I phosphorylation sites. For class II sites, localization probability was between 0.75 and 0.25. Phosphorylation sites with localization probability <0.25 were discarded. The identified phosphopeptides were further processed with MSQuant 1.5 for statistics evaluation as well as quantization.

### Bioinformatics Analysis

Differentially expressed phosphoproteins (DEPPs) were classified based on the PANTHER (Protein ANalysis THrough Evolutionary Relationships) system (http://www.pantherdb.org), which is a unique resource that classifies genes and proteins by their functions [49]. The DEPP interaction network was build automatically by the STRING (Search Tool for the Retrieval of Interacting Genes/Proteins) system with default setting except that organism, confidence(score), and additional (white) nodes were set to “human”, “0.20”, and “10”, respectively[50], [51]. The gene name list of these proteins was input to search against the database which contains known and predicted protein-protein interactions. The retrieve included a detailed network which highlights several hub proteins. The identified phosphoproteins were compared to the public database of PhosphoSite (http://www.phosphosite.org/) to find out the novel phosphoproteins and phosphosites. Each confirmed phosphoprotein was searched with SCANSITE (http://scansite.mit.edu) [52] for potential kinase motifs with high, medium, and low stringency.

### Western Blot Analysis

Protein extracts (30 µg) prepared with RIPA lysis buffer (50 mM Tris-HCl, 150 mM NaCl, 0.1% SDS, 1% NP-40, 0.5% sodium deoxycholate, 1 mM PMSF, 100 mM leupeptin, and 2 mg/mL aprotinin, pH 8.0) were resolved by a 10% SDS-PAGE gel, and transferred onto Immobilon-P PVDF transfer membraneS (Millipore, Bedford, MA) by electroblotting. After blocking with 5% non-fat milk, the membranes were probed with rabbit anti-CaMKII polyclonal, rabbit anti-phospho-CaMKII (Thr286) polyclonal, goat anti-actin polyclonal antibodies (Santa Cruz Biotechnology, Santa Cruz, CA), rabbit anti-CDK2 polyclonal, rabbit anti-phospho-CDK2 (Thr160) polyclonal, rabbit anti-phospho-stathmin (Ser25) polyclonal antibodies (Abcam Inc., Cambridge, MA), rabbit anti-His-tag polyclonal, rabbit anti-stathmin polyclonal, rabbit anti-phospho-stathmin (Ser38) polyclonal, rabbit anti-phospho-stathmin (Ser16) polyclonal, rabbit anti-p44/42 MAPK polyclonal, mouse anti-phospho-p44/42 MAPK (Thr202/Tyr204) monoclonal antibodies (Cell Signaling, Danvers, MA), and mouse anti α-tubulin monoclonal antibodies (DM1A, Sigma, St. Louis, MO). Blots were then incubated with peroxidase-conjugated anti-mouse, anti-rabbit or anti-goat IgG (KPL, Gaithersburg, Maryland) for 1 h at room temperature at a 1∶2000 dilution and then developed by using the SuperSignal West Pico Kit (Pierce Biotechnology, Rockford, IL).

### Generation of Stathmin Mutants

Human stathmin was cloned into the NH_2_ terminal His-tagged pReceiver-M01 expression vector (Genecopoeia, Rockville, MD). Construction of mutant stathmin cDNAs, where the codons for Ser-16, Ser-25 or Ser-38 are exchanged to Ala, was performed by site-directed mutagenesis using a QuikChange kit (Stratagene, La Jolla, CA) following the manufacturer's instructions. Primers used, with the introduced mutations underlined, were: (Ser16→Ala) 5′- AGCGTGCCGCAGGCCAG-3′; (Ser25→Ala) 5′-GCTGATTCTCGCCCCTCGGTC-3′; (Ser38→Ala) 5′-CCCCCTTGCCCCTCCAAAG-3′. All mutant constructs were confirmed by DNA sequence analysis.

### Establishment of Stable Transfectants

The plasmids were introduced into U266 cells using the X005 mode of Nucleofector (Amaxa, Cologne, Germany), according to the Optimized Protocol for the U266B1 cell line. Electroporated cells were cultured in medium with the presence of 0.5 mg/ml G418 (Mediatech, Manassas, VA) for 14 days, and then cultured in the 96-well plates for dilution cloning. Finally, the clone selected from pReceiver-M01 blank vector transfected U266 cells was designated as U266-NC. The clones selected from wild type or mutant stathmin plasmids transfected U266 cells were designated as U266-WT, U266-S16A, U266-S25A or U266-S38A, respectively.

### Assessment of Apoptosis

The extent of apoptosis was evaluated by using Annexin V/PI staining and flow cytometry as described previously [53]. In brief, 1×10^6^ cells were washed once in 1×PBS and were stained with Annexin V-FITC and PI (2 mg/mL) according to manufacturer's instructions. Samples were acquired on a FACScan flow cytometer (Becton Dickinson, San Jose, CA) and analyzed with the WinMDI 2.8 software program.

### Tubulin Polymerization Assay

Tubulin polymerization assay was performed essentially as previously described [29], [54]. Briefly, cells grown to confluency in 24-well plates were washed twice with 1X PBS. To separate polymerized (P) from soluble (S) tubulin, the cells were all incubated at 37°C for 5 min in the dark in hypotonic lysis buffer containing 5 µM paclitaxel, 10 µM Trichostatin-A (Calbiochem, San Diego, CA), 1 mM MgCl2, 2 mM EGTA, 0.5% Nonidet P-40, 2 mM phenylmethylsulfonyl fluoride, 200 units/ml aprotinin, 100 mg/ml soybean trypsin inhibitor, 5.0 mM ε-amino caproic acid, 1 mM benzamidine, and 20 mM Tris-HCl, pH 6.8, vortexed vigorously and centrifuged at ∼15,000 g at 22°C for 10 minutes. The supernatants containing soluble ‘S’ tubulin were transferred to another Eppendorf tube separating them from the pellets containing polymerized ‘P’ tubulin. Upon separation, tubes were placed on ice and pellets of polymerized ‘P’ tubulin were resuspended by sonication for 10-20 seconds in a volume of lysis buffer equal to the soluble ‘S’ fraction. Each had gel sample buffer added, equal aliquots were separated by 10% SDS-PAGE, and western blots using anti-α-tubulin antibody were obtained. The immunoblots were scanned, and densitometric analysis was performed using the public domain NIH Image program ImageJ (available on the Internet at http://rsb.info.nih.gov/nih-image/). The percentage of polymerized ‘P’ tubulin was determined by dividing the densitometry value of polymerized ‘P’ tubulin by the total tubulin content (the sum of the densitometry values of soluble ‘S’ and polymerized ‘P’ tubulin). An advantage of this assay is that the amount of total protein loaded for each sample is irrelevant since the ‘P’ and ‘S’ fractions are equalized for each pair, and it is the proportion of the polymerized to the soluble tubulin fraction that is measured.

### Statistical analysis

All data are expressed as mean ± standard deviation. Statistical significance was determined by Student's *t*-test (two-tailed), while the significance of the differences was determined using the two-tailed Mann–Whitney test. Statistical significance was assigned if *P*<0.05.

## Supporting Information

Data S1 (0.03 MB DOC)

Table S1 List of all identified phosphopeptides and phosphorylation sites.
(0.24 MB XLS)

Table S2 List of all borterzomib regulated phosphoproteins.
(0.03 MB XLS)

Table S3 List of known and novel phosphorylation sites in the identified phosphoproteins.
(0.10 MB XLS)

Table S4 SCANSITE prediction at high stringency (0.2%), medium stringency (1.0%), and low stringency (5.0%) within the identified phosphorylation sites for kinase phosphorylation and binding motifs.
(0.02 MB XLS)

Table S5 The list of phosphorylated site detected according to the kinase.
(0.09 MB XLS)

Table S6 Summary of the growth properties of U266 and derived cells.
(0.03 MB DOC)
