# Supplementary material for: Quantitative Phosphoproteomics of Proteasome Inhibition in Multiple Myeloma Cells
Source: PLoS One. 2010 Sep 29;5(9):e13095. doi: 10.1371/journal.pone.0013095 (PMC2947515; doi:10.1371/journal.pone.0013095)
Supplement: Data S1 — (0.03 MB DOC) [file pone.0013095.s001.doc]

**Supplemental Experimental Procedures**

**Proliferation assay**

To measure cell doubling time, cells were seeded in 24-well plates (3 × 10^5^/well) at day 0, and a hemocytometric count was carried out daily. All measurements were carried out in triplicate. The cell doubling time was determined from the logarithmic growth phase.

**Cell cycle analysis**

The cells were harvested, washed with ice-cold PBS, fixed with 70% ethanol for 1 hour at 4^0^C, and pretreated with RNase (Worthington, Lakewood, NJ) for 30 minutes at 37 ^0^ C. Cells were stained with Propidium iodide (PI) (Sigma Chemical, St. Louis, MO), and cell cycle profile was determined using the FACScan flow cytometer (Becton Dickinson, San Jose, CA). Analysis of the data was done using WinMDI 2.8 software program.

**Measurement of colony-forming efficiency**

Soft agar (1.6% wt/vol) was prepared by autoclaving Bacto-agar (DIFCO, Detroit, MI) in distilled water just before use. The bottom agar layer (2.1 mL/well) contained 1.6% agar: 2×RPMI/20% FBS: 1×RPMI/10% FBS without cells in a volume ratio of 1:1:1, respectively, as a final agar concentration of 0.53%. The top agar layer (0.9 mL/well) contained 1.6% agar: 2×RPMI/20% FBS: 1×RPMI/10% FBS with cells in a ratio 1:1:2, respectively, with a final agar concentration of 0.4%. The number of cells plated for each clone was 5×10^2^/mL. Plates were incubated at 37°C with 5% CO_2_ for 2 weeks and the colonies were stained with Gimsa, and counted under a microscope. The results are the average of three independent experiments.
