# Supplementary material for: Quantitative Phosphoproteomics of Proteasome Inhibition in Multiple Myeloma Cells
Source: PLoS One. 2010 Sep 29;5(9):e13095. doi: 10.1371/journal.pone.0013095 (PMC2947515; doi:10.1371/journal.pone.0013095)
Supplement: Table S6 — Summary of the growth properties of U266 and derived cells. (0.03 MB DOC) [file pone.0013095.s007.doc]

Table S6. Summary of the growth properties of U266 and derived cells.

| **Cell** | **RPMI1640/10% FBS** | **Soft agar** | **Annexin V/ PI staining** |
| --- | --- | --- | --- |
|  | **Doubling G1/S/G2/M**  **Time(hr) Phase, %** | **CFE (%)** | **Apoptotic Ratio (%)** |
| U266 | 33.4 56/30/14 | 23±3.2 | 4.2±1.6% |
| U266-NC | 35.6 54/30/16 | 24±2.1 | 3.6±1.2% |
| U266-WT | 37.2 55/31/14 | 26±5.2 | 5.4±1.4% |
| U266-S16A | 32.6 53/26/21 | 28±3.7 | 3.2±1.6% |
| U266-S25A | 31.8 54/28/18 | 22±4.2 | 6.2±2.1% |
| U266-S38A | 32.9 50/30/20 | 21±3.9 | 5.0±2.1% |

CFE, colony-forming efficiency; FBS, fetal bovine serum
